# Outbreak of Pneumonia in the Setting of Fatal Pneumococcal Meningitis among US Army Trainees: Potential Role of *Chlamydia pneumoniae *Infection

**DOI:** 10.1186/1471-2334-11-157

**Published:** 2011-06-02

**Authors:** Fatimah S Dawood, John F Ambrose, Bruce P Russell, Anthony W Hawksworth, Jonas M Winchell, Nina Glass, Kathleen Thurman, Michele A Soltis, Erin McDonough, Agnes K Warner, Emily Weston, Nakia S Clemmons, Jennifer Rosen, Stephanie L Mitchell, Dennis J Faix, Patrick J Blair, Matthew R Moore, John Lowery

**Affiliations:** 1 Epidemic Intelligence Service, Office of Workforce and Career Development assigned to Influenza Epidemiology and Prevention Branch, Influenza Division, Centers for Disease Control and Prevention, 1600 Clifton Road NE, Atlanta, Georgia, 30333, USA; 2 U.S. Army Center for Health Promotion and Preventive Medicine, 5158 Blackhawk Road, Aberdeen Proving Ground, Maryland, 21010, USA; 3 General Leonard Wood Army Community Hospital, Preventive Medicine Division, 126 Missouri Avenue, Fort Leonard Wood, Missouri, 65473, USA; 4 Naval Health Research Center, PO Box 85122, San Diego, California, 92106, USA; 5 Respiratory Diseases Branch, Division of Bacterial Diseases, National Center for Immunization and Respiratory Diseases, Centers for Disease Control and Prevention, 1600 Clifton Road NE, Atlanta, Georgia, 30333, USA; 6 General Leonard Wood Army Community Hospital, 126 Missouri Avenue, Fort Leonard Wood, Missouri, 65473, USA

**Keywords:** Pneumonia, pneumococcal, Chlamydophila, pneumoniae, Military Personnel

## Abstract

**Background:**

Compared to the civilian population, military trainees are often at increased risk for respiratory infections. We investigated an outbreak of radiologically-confirmed pneumonia that was recognized after 2 fatal cases of serotype 7F pneumococcal meningitis were reported in a 303-person military trainee company (Alpha Company).

**Methods:**

We reviewed surveillance data on pneumonia and febrile respiratory illness at the training facility; conducted chart reviews for cases of radiologically-confirmed pneumonia; and administered surveys and collected nasopharyngeal swabs from trainees in the outbreak battalion (Alpha and Hotel Companies), associated training staff, and trainees newly joining the battalion.

**Results:**

Among Alpha and Hotel Company trainees, the average weekly attack rates of radiologically-confirmed pneumonia were 1.4% and 1.2% (most other companies at FLW: 0-0.4%). The pneumococcal carriage rate among all Alpha Company trainees was 15% with a predominance of serotypes 7F and 3. *Chlamydia pneumoniae *was identified from 31% of specimens collected from Alpha Company trainees with respiratory symptoms.

**Conclusion:**

Although the etiology of the outbreak remains unclear, the identification of both *S. pneumoniae *and *C. pneumoniae *among trainees suggests that both pathogens may have contributed either independently or as cofactors to the observed increased incidence of pneumonia in the outbreak battalion and should be considered as possible etiologies in outbreaks of pneumonia in the military population.

## Background

Military trainees are at increased risk for respiratory infections compared to the general civilian population[1]. Crowded living conditions and intense physical stress may contribute to an increased risk for infections with *Streptococcus pneumoniae, Streptococcus pyogenes, Mycoplasma pneumoniae, Chlamydia pneumoniae, Bordetella pertussis*, adenoviruses, and influenza viruses[1-3]. Outbreaks of pneumococcal disease are relatively rare in the general civilian population, but sporadic outbreaks of pneumococcal pneumonia have been reported among military trainees in the past[4-8]. We describe the investigation of an outbreak of radiologically-confirmed pneumonia in the setting of two fatal cases of pneumococcal meningitis that occurred in February, 2009, at a Fort Leonard Wood, MO military training facility. The findings of this investigation provide information about the epidemiology and microbiology of outbreaks of acute respiratory illness in military training populations and may inform military health policy.

## Methods

### Setting

During February 6-14, 2009, two previously healthy military trainees were diagnosed with *S. pneumoniae *meningitis and subsequently died. Both trainees belonged to the same company (Alpha Company, n = 303) in the 554^th ^Battalion at Fort Leonard Wood U.S. Army Maneuver Support Center of Excellence (FLW) training camp in Missouri. During the same period of time (from February 6-14, 2009), an increase in the number of cases of radiologically-confirmed pneumonia was also identified at FLW training camp.

FLW training camp has a trainee population that typically ranges from 12,000-15,000 trainees during the winter months, with new trainees entering and graduating from the camp every two weeks. Trainees are sent to FLW training camp to receive Initial Entry Training (IET), Advanced Individual Training (AIT), or both. Trainees undergoing IET belong to separate battalions and reside in separate living quarters than trainees undergoing AIT. In total, there are 13 battalions that are further subdivided into 33 companies. Each battalion has its own associated training staff, some of whom work closely with trainees despite living in separate training staff quarters. Trainees arriving for IET at Fort Leonard Wood receive antibiotic chemoprophylaxis with benzathine penicillin G during their initial in-processing. However, trainees transferring from other basic training sites for AIT do not receive antibiotic chemoprophylaxis upon arrival at FLW training camp, regardless of whether or not they received prior chemoprophylaxis. In addition, 23-valent pneumococcal polysaccharide vaccine (PPV23) is administered only to the small minority of individuals with underlying illnesses that increase risk for pneumococcal disease[9]. All trainees are required to receive annual influenza vaccination.

Trainees of the 554^th ^Battalion are assigned to either Alpha or Hotel Companies to undergo AIT in heavy machinery operation. Alpha and Hotel companies are housed separately and train separately.

### Case definitions, case ascertainment, and surveillance review

A case of pneumococcal meningitis was defined as a trainee or training staff member in Alpha or Hotel Company with symptoms consistent with meningitis and growth of *S. pneumoniae *from cerebrospinal fluid during February 1-21, 2009. Cases were identified by review of hospital records at FLW training camp and by referral from civilian hospitals.

A case of radiologically-confirmed pneumonia was defined as a trainee or training staff member in Alpha or Hotel Company with a new infiltrate identified on chest radiograph by the treating clinician or a radiologist from February 1-21, 2009. Cases were identified through existing General Leonard Wood Army Community Hospital (GLWACH) surveillance for radiologically-confirmed pneumonia, which was conducted by reviewing the weekly list of trainees deemed by a physician to be too ill to participate in routine training and reviewing the electronic medical records of trainees on this list for documentation of radiologically-confirmed pneumonia. Using a standardized data abstraction form, we reviewed the medical charts of all patients with radiologically-confirmed pneumonia from companies with elevated rates of pneumonia, and we collected data on symptoms of illness^1^, past medical history, laboratory and diagnostic testing, pertinent exam findings, and recent treatment with antimicrobial medications.

The Naval Health Research Center (NHRC) conducts surveillance year-round among trainees at FLW training camp and seven other Department of Defense recruitment training camps for febrile respiratory illness (FRI), defined as temperature≥100.5°F with cough or sore throat. Trainees that meet the criteria for FRI are informed about the research volunteer program. Trainees that volunteer to participate have nasal and throat swabs collected and forwarded to NHRC where they are routinely tested by polymerase chain reaction (PCR) for adenovirus and influenza A virus and by culture-based assay for adenovirus, enterovirus, herpes simplex virus, parainfluenza 1-3, influenza A and B viruses, and respiratory syncytial virus (RSV). We reviewed FRI surveillance data from FLW training camp from January 1-February 17, 2009. During February 1-14, 2009, 17 nasal and throat swabs were collected from FLW training camp trainees and tested as part of routine FRI surveillance; none of these were from Alpha or Hotel Company trainees. During February 15-16, 2009, GLWACH opened a special clinic for trainees in the 554^th ^Battalion who were ill with respiratory symptoms. Of 65 trainees from the 554^th ^Battalion seen at the special clinic, a convenience sample of 37 trainees (57%) was selected to have nasal and throat swab specimens collected; these specimens were sent to NHRC where they were tested by PCR for mimivirus, bocavirus, *Chlamydia pneumoniae, Mycoplasma pneumoniae, Streptococcus pneumoniae, Bordetella pertussis*, and *Legionella pneumophila*[10] in addition to undergoing testing for pathogens included in routine FRI surveillance testing.

### Cross-sectional survey

To assess the burden of respiratory illness during the investigation period (February 1-21, 2009), we administered a standardized survey to all trainees and training staff of the 554^th ^Battalion. The survey collected demographic information and information on symptoms of illness, healthcare utilization, influenza vaccination status, and treatment with antibiotics during February 1, 2009 to the time of survey administration (February 18-21, 2009). As a comparison group, we also administered the same survey asking about the same time period to all incoming Alpha Company trainees in the 554^th ^Battalion on the day they arrived at the FLW training camp from their homes in the civilian community.

To assess circulation of the pneumococcal strain that caused the two cases of meningitis, we conducted a cross-sectional survey of all Alpha company trainees, training staff members, incoming trainees to the Alpha Company, and, because of limited supplies, only the first 25 members of the Hotel Company. We collected calcium alginate nasopharyngeal swabs, stored them in skim milk, tryptone, glycerol, and glucose [11], and shipped them overnight to CDC's Streptococcal Reference Laboratory. The skim milk, tryptone, glycerol, and glucose media containing the NP samples were briefly thawed and homogenized and 200 μl were inoculated in 5.0 ml of Todd Hewitt broth containing 0.5% yeast extract supplemented with 1 ml of rabbit serum. After 4-6 hours of CO2 incubation at 37°C, 10 μl of the broth were inoculated into blood-agar plates and incubated overnight. *S. pneumoniae *colonies were identified morphologically and confirmed using bile solubility and optochin susceptibility. Confirmed isolates were serotyped by Quellung reaction.

To assess the potential roles of other pathogens in the outbreak, we obtained nasopharyngeal swabs and throat swabs for viral and atypical bacterial pathogen testing from Alpha and Hotel company trainees and incoming Alpha company trainees with self-reported fever and two or more respiratory symptoms within the preceding 72 hours. We also obtained an additional nasopharyngeal swab and throat swab for viral and atypical bacterial pathogen testing from some symptomatic trainees who did not meet these criteria. Throat swabs were collected from all symptomatic and asymptomatic Alpha and Hotel Company training staff members. Nasopharyngeal swabs were tested for influenza A and B, RSV, parainfluenza 1-3, human metapneumovirus (hMPV), adenovirus, [12] and rhinovirus [12] by real-time PCR. Throat swabs were tested for *C. pneumoniae*[13] and *M. pneumoniae*[14] by real-time PCR.

### Human Subjects Review

The purpose of this investigation was to identify the cause of the outbreak and to implement interventions to prevent further cases from occurring. As such, the investigation was not considered to be research and was therefore exempt from human subjects review.

### Statistical Analysis

Survey data were entered into a Microsoft Access 2003 database and analyzed using SAS 9.1 (SAS Institute Inc., Cary, NC). Chi-square and odds ratios with 95% confidence intervals were calculated for trainee and training staff characteristics hypothesized to be associated with carriage or detection of pertinent pathogens.

Using denominators from the battalion, company, and training staff rosters, weekly attack rates were calculated for each battalion and company at FLW training camp and for the training staff of the 554^th ^Battalion.

## Results

### Case-ascertainment and surveillance for meningitis, radiologically-confirmed pneumonia, and FRI

**Figure 1:**
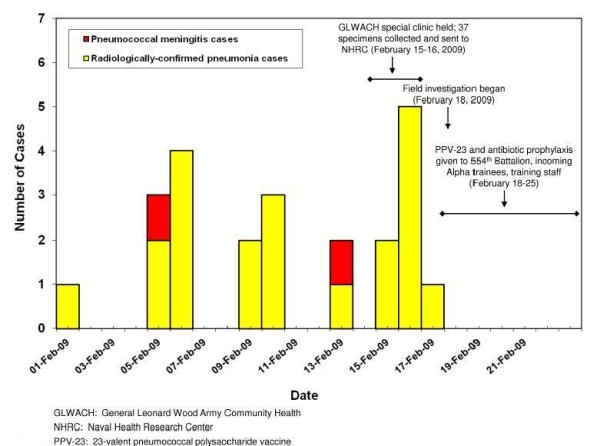
**Cases of pneumococcal meningitis and radiologically-confirmed pneumonia in the 554th Battalion, Fort Leonard Wood, MO, February 1-21, 2009**.

The two cases of pneumococcal meningitis were the only meningitis cases identified during the investigation period, and both case-patients died (figure 1). Both case-patients had confirmed infection with pneumococcal serotype 7F which was fully susceptible to penicillin (minimum inhibitory concentration ≤ 0.06 mcg/ml). For both patients, blood cultures were negative, sputum cultures revealed normal flora, and there was no evidence of pneumonia on chest radiographs. Autopsies were not performed.

Seventy-two cases of radiologically-confirmed pneumonia were identified among all trainees (N = approximately 9,400; approximate average weekly attack rate 0.3%) at FLW training camp during February 1-February 21. Of those 72 cases, 21 (29%) occurred among trainees in the 554th Battalion (figure 1); 11 cases occurred in Alpha Company (average weekly attack rate 1.4%), and 10 cases occurred in Hotel Company (average weekly attack rate 1.2%). Of the 21 identified cases in the Battalion, one (5%) was hospitalized, four (19%) had sputum cultures sent at the time of diagnosis, and three (14%) had blood cultures sent to the GLWACH laboratory at the time of diagnosis; all sputum and blood cultures were negative. None of the cases had urinary antigen testing for *S. pneumoniae*. All 21 cases had chest radiographs interpreted by the reviewing radiologist as having evidence of lobar or lingular pneumonia. Of five trainees with pneumonia from whom nasal and throat swabs were obtained on the day of diagnosis as part of the special clinic held by GLWACH for Alpha and Hotel company trainees, *C. pneumoniae *was identified by PCR from four (80%); testing for other pathogens including influenza viruses was negative.

During February 18-19, 2009, eleven of the Alpha and Hotel Company trainees with radiologically-confirmed pneumonia already treated with antibiotics had nasopharyngeal swab specimens sent to CDC for *S. pneumoniae *testing; of these, *S. pneumoniae *was isolated from specimens from two trainees (18%; one serotype 7F, one serotype 3). Seven Alpha and Hotel Company trainees with radiologically-confirmed pneumonia already treated with antibiotics had nasopharyngeal and throat swab specimens sent to CDC for viral and atypical pathogen testing; of these, *C. pneumoniae *was identified in specimens from two (29%) trainees.

FRI was not increased at FLW training camp from February 1-February 17, 2009. Of the 17 FRI specimens collected from FLW training camp basic trainees during this time as part of routine FRI surveillance, 6 (35%) were positive for influenza A and none were positive for *C. pneumoniae*.

### Survey

Of the 533 trainees (99.6%) from the 554^th ^Battalion who completed an investigation survey, the median age was 22 years (range 17-51) and 20 (4%) had an underlying medical condition (table 1). Most trainees (67%) lived in "bays" (defined as rooms occupied by more than four trainees). Only 28 (5%) trainees reported having received PPV23 in the past, and of these, 16 (57%) had an indication for PPV23 (two had asthma and 14 were current smokers)^2^. Receipt of PPV23 that occurred before entering military service was not always documented in military medical records. Therefore, self-reported pneumococcal vaccination status could not be verified with military medical records.

**Table 1:** Characteristics and symptoms of Alpha and Hotel Company trainees, battalion training staff, and incoming trainees (N = 713)

Characteristic	Alpha Company	Hotel Company	Training Staff	Incoming Alpha
	**n = 301**	**n = 232**	**n = 131**	**n = 49**

**Median age (years)**	22	22	37	22
**Male**	290 [96%]	213 [92%]	126 [96%]	49 [100%]
**Underlying medical condition**	6 [2%]	14 [6%]	21 [16%]	1 [2%]
**Smoking status**				
Smoked > 100 cigarettes in lifetime	185 [61%]	146 [63%]	75 [57%]	38 [78%]
Currently smoke	163 [54%]	72 [31%]	50 [38%]	13 [27%]
**Living quarters**				
Bay (> 4 trainees per room)	209 [69%]	148 [64%]	N/A	N/A
Smaller room (2-4 trainees per room)	90 [30%]	86 [37%]	N/A	N/A
**Received 2008-09 influenza vaccine^a,b^**	271/276 [98%]	216/219 [99%]	92/122 [75%]	36/38 [95%]
**Received pneumococcal vaccine^c^**	18 [6%]	10 [4%]	3 [2%]	6 [12%]
**Symptoms February 1-19, 2009**				
At least 1 symptom^d^	247 [82%]	183 [79%]	77 [59%]	23 [47%]
Cough	211 [70%]	156 [67%]	53 [40%]	12 [24%]
Runny Nose	195 [65%]	153 [66%]	53 [40%]	18 [37%]
Sore Throat	125 [42%]	82 [35%]	38 [29%]	7 [14%]
Fever	61 [20%]	45 [19%]	17 [13%]	2 [4%]
Chills	53 [18%]	39 [17%]	9 [7%]	0
**Received antibiotics February 1-19, 2009**	87 [29%]	62 [27%]	18 [14%]	5 [10%]

*^a^*An individual was considered vaccinated against influenza for the current season if the individual received the 2008-09

influenza vaccine > 14 days prior to participation in the cohort investigation

^b^Of individuals who reported knowing their influenza vaccination status for the current season

^c^Self-reported history of receipt of pneumococcal vaccine

^d^Of the following symptoms: fever, chills, cough with or without sputum, difficulty breathing, wheezing, runny nose, sore throat, or ear pain

Of the 533 surveyed Alpha and Hotel Company trainees, only 103 (19%) did not have self-reported fever, chills, or respiratory symptoms (defined as cough with or without sputum, difficulty breathing, wheezing, runny nose, sore throat, or ear pain) during February 1, 2009, through the date of survey on February 18-19, 2009. Of the 430 (81%) trainees with at least one of these symptoms, cough was the most common symptom (69%), followed by runny nose (66%) and sore throat (39%). Alpha and Hotel Company trainees were significantly more likely to report symptoms compared to the incoming Alpha Company trainees (OR 5.2, CI 2.9-9.3) and to training staff (OR 3.5, CI 2.4-5.3). Among surveyed trainees, 149 (27%) trainees received antibiotics from February 1-19, 2009.

**Figure 2:**
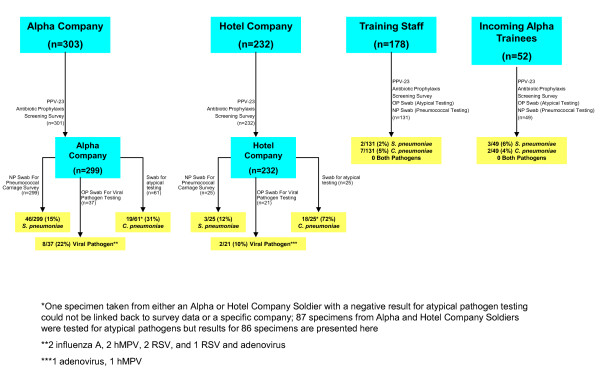
**Respiratory pathogen testing among Alpha and Hotel Company trainees, training staff, and incoming Alpha Company trainees, Fort Leonard Wood, MO, February 2009**.

Of 299 specimens tested from Alpha Company trainees, *S. pneumoniae *was isolated from 46 specimens (15%) (figure 2). Of those 46 isolates, 45 isolates were typeable (including 1 isolate containing 2 serotypes) and 10 serotypes were identified: 14 (30%) serotype 7F; 14 (30%) serotype 3; 6 (13%) serotype 23A, 5 (11%) serotype 7C, 2 (4%) serotype 17F, and 1 (2%) each of serotypes 11A, 15A, 16F, 35F, and 38.

Specimens were obtained for pneumococcal testing from 25 (12%) Hotel Company trainees. *S. pneumoniae *was isolated from three specimens (overall carriage 12%). Of those three isolates, two were serotype 19A, and one was serotype 1.

Of the 49 specimens obtained from incoming Alpha Company trainees, *S. pneumoniae *was isolated from three specimens (6%). Of those three isolates, two were serotype 17F, and one was serotype 22F. Of the 131 specimens obtained from battalion training staff, *S. pneumoniae *was isolated from two specimens (2%): one serotype 11A, and one serotype 35B.

*C. pneumoniae *was identified in 19 of 61 (31%) specimens obtained from Alpha Company trainees, 18 of 25 (72%) from Hotel Company trainees, 7 of 131 (5%) from battalion training staff, and 2 of 49 (4%) from incoming Alpha trainees. Of the 46 individuals with specimens positive for *C. pneumoniae*, all had respiratory symptoms and/or self-reported fever within the two weeks preceding swabbing.

Of the 37 specimens from Alpha Company trainees tested for viral respiratory pathogens, viral pathogens were identified from eight (22%) specimens: two with influenza A, two with hMPV, two with RSV, and one with both RSV and adenovirus. Of the 21 specimens from Hotel Company, viral pathogens were identified from two (10%) specimens: one with adenovirus and one with hMPV.

Table 2 summarizes factors that were explored for potential association with pneumococcal carriage and *C. pneumoniae *detection. Taking antibiotics, living in a "bay," current smoking, and living on a particular floor of the barracks were not significantly associated with pneumococcal carriage or *C. pneumoniae *detection. In addition, having one or more illness symptoms was not significantly associated with pneumococcal carriage.

**Table 2:** Factors associated with pneumococcal carriage or *C. pneumoniae *detection among Alpha and Hotel Company trainees

	*S. pneumoniae *positive	*S. pneumoniae *negative	OR (CI)
	n = 49	n = 275	

Receipt of antibiotics^a^	7/37 (19%)	82/240 (34%)	0.4 (0.2-1.1)
"Bay" resident (> 4 trainees per room)	35 (71%)	191 (69%)	1.1 (0.6-2.2)
Current smoker	30 (61%)	142 (52%)	1.5 (0.8-2.8)
Floor of barracks			
Floor 1	7/42 (17%)	64/233 (28%)	0.6 (0.2-1.6)
Floor 2	20/42 (47%)	87/233 (37%)	1.3 (0.6-2.6)
Floor 3	15/42 (36%)	82/233 (35%)	1 (Ref)^b^
One or more symptoms^c^	35 (71%)	229 (83%)	0.5 (0.3-1.0)

	***C. pneumoniae *****positive**	***C. pneumoniae *****negative^d^**	**OR (CI)**
	n = 37	n = 48	

Receipt of antibiotics^a^	25 (69%)	34 (71%)	0.9 (0.3-2.3)
"Bay" resident (> 4 trainees per room)	30 (81%)	31 (65%)	2.4 (0.9-6.5)
Current smoker	18 (49%)	16 (33%)	1.9 (0.8-4.6)
Floor of barracks			
Floor 1	2/34 (6%)	9/45 (20%)	0.2 (0-1.0)
Floor 2	11/34 (32%)	15/45 (33%)	0.6 (0.2-1.7)
Floor 3	21/34 (62%)	18/45 (40%)	1 (Ref)^b^

^a^Trainee reported taking antibiotics during February 1-19, 2009

^b^Trainees living on the 3rd floor of the barracks were used as the reference group for comparison with trainees living on the first and second floors of the barracks

^c^Of the following symptoms: fever, chills, cough with or without sputum, difficulty breathing, wheezing, runny nose, sore throat or ear pain

^d ^Two Alpha Company trainees with respiratory specimens that were negative for *C. pneumoniae *were not included in univariate analysis including one trainee who had a sample that could not be linked back to the survey data and one trainee who gave a respiratory specimen but did not complete a survey

### Interventions

From February 18-25, 2009, 301 of the 303 trainees currently in the Alpha Company, all 232 trainees in the Hotel Company, 131 of the 178 battalion training staff (i.e. those who opted to participate in the investigation), and 49 of 52 incoming trainees to the Alpha Company received 23-valent pneumococcal polysaccharide vaccine (PPV23) and antibiotic prophylaxis (benzathine penicillin G 1.2 million units given intramuscularly or azithromycin 1 gram given orally for those with penicillin allergy). In addition, hand hygiene and cough etiquette were reviewed with all trainees and battalion training staff.

## Discussion

We describe an outbreak of radiologically-confirmed pneumonia that was first recognized in a military training company after two fatal cases of pneumococcal meningitis caused by serotype 7F occurred in the same company. Overall, 15% of trainees in the 554^th ^Battalion were colonized with pneumococcus, and serotypes 7F and 3 were predominant. Colonization rates of 14-44% have been documented among military personnel in the setting of other pneumococcal outbreaks[4,7,8]. Because pneumococcus is the most common bacterial cause of pneumonia and meningitis in the United States, *C. pneumoniae *detection among 44% of trainees tested was unexpected and suggests a possible outbreak of *C. pneumoniae *infection in the same battalion.

Most adults with pneumonia, whether civilian or military, do not undergo etiologic testing for pneumonia. Our investigation began after 21 trainees in the 554^th ^Battalion had already been diagnosed with pneumonia and treated with antibiotics. Thus, the etiology of their pneumonia remains unclear. Pneumococcus has been documented as the primary etiologic agent in several outbreaks of pneumonia among military trainees described in the literature[1,4-6,8,15]. During this outbreak of pneumonia at FLW training camp, the isolation of pneumococcus serotype 7F from the CSF of the two trainees with fatal meningitis and the predominance of serotypes 7F and 3 among trainees from their battalion may indicate that increased pneumococcal transmission was occurring at the time of the outbreak and provides some indirect support for pneumococcus as a possible etiologic agent or cofactor in the outbreak of pneumonia. However, blood cultures were obtained at the time of pneumonia diagnosis from only three trainees with pneumonia, all of whom had cultures with no growth, and pneumococcal urinary antigen testing was not performed for any of the trainees with pneumonia.

*C. pneumoniae *may have also played a role in this outbreak of pneumonia, either as a primary etiologic agent or as a cofactor, as suggested by the increased prevalence of *C. pneumoniae *identification among trainees in the battalion compared to training staff and incoming trainees. *C. pneumoniae *is recognized as a common cause of community acquired pneumonia in young adults, and two outbreaks of pneumonia associated with *C. pneumoniae *have been documented among military recruits in Europe[3,16]. One study conducted in a Finnish civilian population also suggested that *C. pneumoniae *infection can predispose individuals to subsequent infection with *S. pneumoniae*[17].

Every year, approximately 68,500 individuals participate in training at FLW training camp, and typically 12,000-15,000 trainees are at the training camp at any given time. The constant turn-over of trainees at military training facilities poses a challenge for timely rate-based surveillance at the company level because the number of trainees at risk changes every one to two weeks. In this investigation, the outbreak of pneumonia among Alpha and Hotel Company trainees was identified by the GLWACH medical command, in part because of the two fatal cases of pneumococcal meningitis, as well as through GLWACH's surveillance for radiologically-confirmed pneumonia. To facilitate outbreak detection and response, including antibiotic prophylaxis and vaccination when indicated, on-going, timely analysis of surveillance data at the company level at military training installations should be considered.

PPV23 and antibiotic prophylaxis, primarily with benzathine penicillin G, were used in an attempt to halt this outbreak because the serotype that caused the two cases of meningitis is included in PPV23 and was susceptible to penicillin. In the absence of a control group with similarly high attack rates of pneumonia, we were unable to determine the effectiveness of PPV23 or chemoprophylaxis in preventing further cases of pneumonia or meningitis. PPV23 and antibiotic prophylaxis have previously been used in other clusters of severe pneumococcal infection [18] including in military settings[4,6]. Since most PPV23 recipients require two weeks to develop immunity after vaccination, antibiotic prophylaxis has been used as an adjunct in outbreak settings to provide additional protection until immunity has developed. Antibiotic prophylaxis against pneumococcus can also decrease nasopharyngeal colonization with pneumococcus and decrease pneumococcal transmission.

Pneumococcal vaccination is a proven method of prevention of pneumococcal disease. Vaccination with PPV23 was provisionally recommended for smokers and persons with asthma by the Advisory Committee on Immunization Practices (ACIP) at the time of this investigation and is now officially recommended for these groups by the ACIP[9]. The Military Vaccine Agency does not recommend vaccination with PPV23 for all military trainees but does offer the vaccine to those considered at high risk of pneumococcal infection. The potential licensure of pneumococcal conjugate vaccines for adults in the United States may benefit military trainees because such vaccines prevent colonization with and transmission of vaccine-type pneumococci. The advent of pneumococcal common protein vaccines, which would provide protection against all serotypes of *S. pneumoniae*, may offer another opportunity for prevention of pneumococcal disease among military trainees in the future[19].

Our investigation was subject to at least two limitations. First, since our investigation began after the majority of pneumonia cases had occurred in the 554^th ^Battalion, we relied, in part, on diagnostic tests performed by the treating clinician at the time of pneumonia diagnosis to try to identify the etiology of the outbreak of pneumonia, and these diagnostic tests were not performed in a systematic manner. Second, since this investigation was undertaken as part of a public health response, we could not survey and obtain respiratory specimens from an unaffected training company with a lower pneumonia attack rate for comparison to trainees in the Alpha and Hotel companies. Inclusion of such a comparison group would have provided data on pneumococcal carriage and *C. pneumoniae *detection rates among trainees with a lower rate of pneumonia who were living and training under similar condition as Alpha and Hotel company trainees.

## Conclusions

We describe an outbreak of radiologically-confirmed pneumonia and two fatal cases of pneumococcal meningitis that occurred among military trainees and the interventions that were made to halt the outbreak. Because etiologic testing was not conducted among the trainees with pneumonia at the time of pneumonia diagnosis, the etiology of the outbreak remains unclear. However, *S. pneumoniae *and *C. pneumoniae *may have both contributed independently or as cofactors to the burden of respiratory illness observed among trainees. Rate-based surveillance for pneumonia at the company-level on military training installations may improve outbreak detection and timely response. In addition, new pneumococcal vaccines may provide opportunities to reduce the burden of pneumococcal disease among military trainees in the future.

## Abbreviations

(FLW): Fort Leonard Wood U.S. Army Maneuver Support Center of Excellence; (IET): Initial Entry Training; (AIT): Advanced Individual Training; (PPV-23) 23-valent pneumococcal polysaccharide vaccine; (GLWACH): General Leonard Wood Army Community Hospital; (NHRC): Naval Health Research Center; (FRI): Febrile Respiratory Illness; (PCR): polymerase chain reaction; (RSV): respiratory syncytial virus; (hMPV): human metapneumovirus.

## Competing interests

The authors declare that they have no competing interests.

## Authors' contributions

FD, JA, BR, NG, MS, EW, NC, JR, DF, PB, MM, and JL participated in the design and conduct of the field investigation. AH, JW, KT, EM, AW, and SM carried out the laboratory testing of respiratory specimens for viral and atypical pathogens. FD and EW analyzed the data. FD and MM drafted the manuscript. All authors read and approved the final manuscript.

## Endnotes

^1 ^Defined as self-reported fever, chills, cough with or without sputum, difficulty breathing, wheezing, runny nose, sore throat, or ear pain.

^2 ^Vaccination with PPV23 was provisionally recommended for smokers and persons with asthma by the Advisory Committee on Immunization Practices (ACIP) at the time of this investigation.

## Pre-publication history

The pre-publication history for this paper can be accessed here:

http://www.biomedcentral.com/1471-2334/11/157/prepub
